# A socio-ecological model of factors influencing physical activity in pregnant women: a systematic review

**DOI:** 10.3389/fpubh.2023.1232625

**Published:** 2023-11-20

**Authors:** Junjiang Sun, Magdalena Piernicka, Aneta Worska, Anna Szumilewicz

**Affiliations:** ^1^Faculty of Physical Culture, Gdansk University of Physical Education and Sport, Gdansk, Poland; ^2^Higher Vocational College, Yunnan College of Business Management, Kunming, China

**Keywords:** physical activity, influencing factors, pregnant women, social–ecological model, systematic review, pregnancy

## Abstract

Physical activity (PA) is safe for most pregnant women, improving both maternal fitness and birth outcomes. Despite evidence of benefits, most pregnant women eliminate or reduce PA during pregnancy. This systematic review aimed to analyze the factors affecting maternal PA during pregnancy with reference to a socio-ecological model. A systematic search of relevant published studies between 2001 and 2022 was conducted through PubMed, Scopus, Web of Science, Academic Search Ultimate, Medline, and SPORTDiscus with full text via the EBSCO platform. A total of 32 studies that met the inclusion criteria were reviewed. The findings revealed that various study designs can lead to different outcomes in terms of what is identified as a PA facilitator or barrier. The factors that positively influenced PA in pregnant women were: higher levels of education, knowledge, and skills, as well as access to mass media. Conversely, lower levels of education, lack of knowledge and skills, low income, pregnancy discomforts, limited time, safety concerns, and societal perceptions of PA in pregnancy acted as barriers. Additionally, family, colleagues/friends, and partners could either support or hinder PA. Factors affecting overall maternal PA were somewhat different from those affecting the moderate-to-vigorous intensity of PA. Pregnant women receive little organizational and policy support. There is an urgent need to provide accessible information and resource systems for pregnant women. Since most pregnant women are motivated to engage in PA and susceptible to family advice, interventions should not be limited only to pregnant women, but should involve a family member, especially partners. There is a need to take global, systemic actions to promote an active lifestyle in pregnancy. Addressing safety concerns related to PA during pregnancy should be a significant part of these promotional activities.

## 1 Introduction

Physical activity (PA) refers to any bodily movement produced by skeletal muscles that require energy expenditure, including activities undertaken while working, playing, doing household chores, traveling, and engaging in recreational activities (1, 2). Current guidelines published by credible obstetrics, gynecology, and sports medicine institutions, including the World Health Organization (WHO), confirm PA in pregnancy is safe and desirable in the absence of obstetric and medical complications or contraindications (3–6). During pregnancy, proper and sufficient PA plays a significant role in the health of the mother and the growth of the fetus (7, 8), including decreasing the incidence of preterm birth (9) and cesarean deliveries (10), avoiding excessive gestational weight gain (11), improving cardiovascular function (12), improving or maintaining physical fitness, reducing symptoms of depression (13), and enhancing psychological well-being (14). Nevertheless, many women tend to decrease rather than maintain or increase their PA during pregnancy (15, 16), and various studies indicate low levels of PA among pregnant women (17, 18).

Exercise is a subset of physical activity that is planned, structured, and repetitive and has as a final or an intermediate objective the improvement or maintenance of physical fitness. Exercise-related behavior is multifaceted and affected by many factors to varying degrees, which makes it complex to engage in PA (19). The PA of pregnant women is also affected by a variety of factors (20), so it is important to know which main factors are associated with PA behavior. A previous literature search found that most of the research on the PA of pregnant women focused on lifestyle interventions, and there were very few reviews on influencing factors of the PA of pregnant women based on a socio-ecological model. Consequently, the main aim of this review is to analyze the influencing factors of maternal PA in a socio-ecological model. We also aimed at exploring the disparities in influencing factors between overall PA (which refers to all kinds of bodily movements of varying intensities, including very low and low intensities) and moderate-to-vigorous physical activity (MVPA) among pregnant women. This will provide a reference for the research, intervention, and policy development to support the promotion of maternal PA.

## 2 The socio-ecological model

The PA of pregnant women is affected by a variety of factors (20). The multifactorial health promotion was advocated in the Ottawa Charter for Health Promotion as early as 1986 (21). For a more comprehensive understanding of the factors affecting the PA of pregnant women using the socio-ecological model (SEM) in line with McLeroy et al. (22), behavior is viewed as being determined by the following levels: (1) the personal level: the internal factors of individual characteristics (sociodemographic and biological, behavioral, psychological); (2) the interpersonal level: interpersonal processes and primary groups – formal and informal social networks and social support systems (e.g., family, public, etc.); (3) the organizational level: social institutions with organizational characteristics, such as health services and gyms, may also include influences from health care providers and PA consultants, etc.; (4) the community level: relationships among organizations, institutions, and informal networks within defined boundaries (e.g., appropriate facilities, living environment, etc.); and finally (5) the public policy level: local, state, and national laws and policies.

## 3 Materials and methods

The systematic review was conducted using “The PRISMA 2020 statement: An updated guideline for reporting systematic reviews” for the analysis material (23). The study protocol was registered on INPLASY (Registration number: INPLASY2022.11.0073). Bibliographic platforms and databases were searched, including PubMed, Scopus, Web of Science, Academic Search Ultimate, Medline, and SPORTDiscus with full text via the EBSCO search platform. The time range was set to 2001–2022, using the terms (“physical activity” or “exercise” or “fitness “or “physical exercise” or “sport”; “correlates” or “determinants” or “mediators” or “associated factors” or “psychosocial” or “environment”; “pregnant women” or pregnancy).

**Figure 1:**
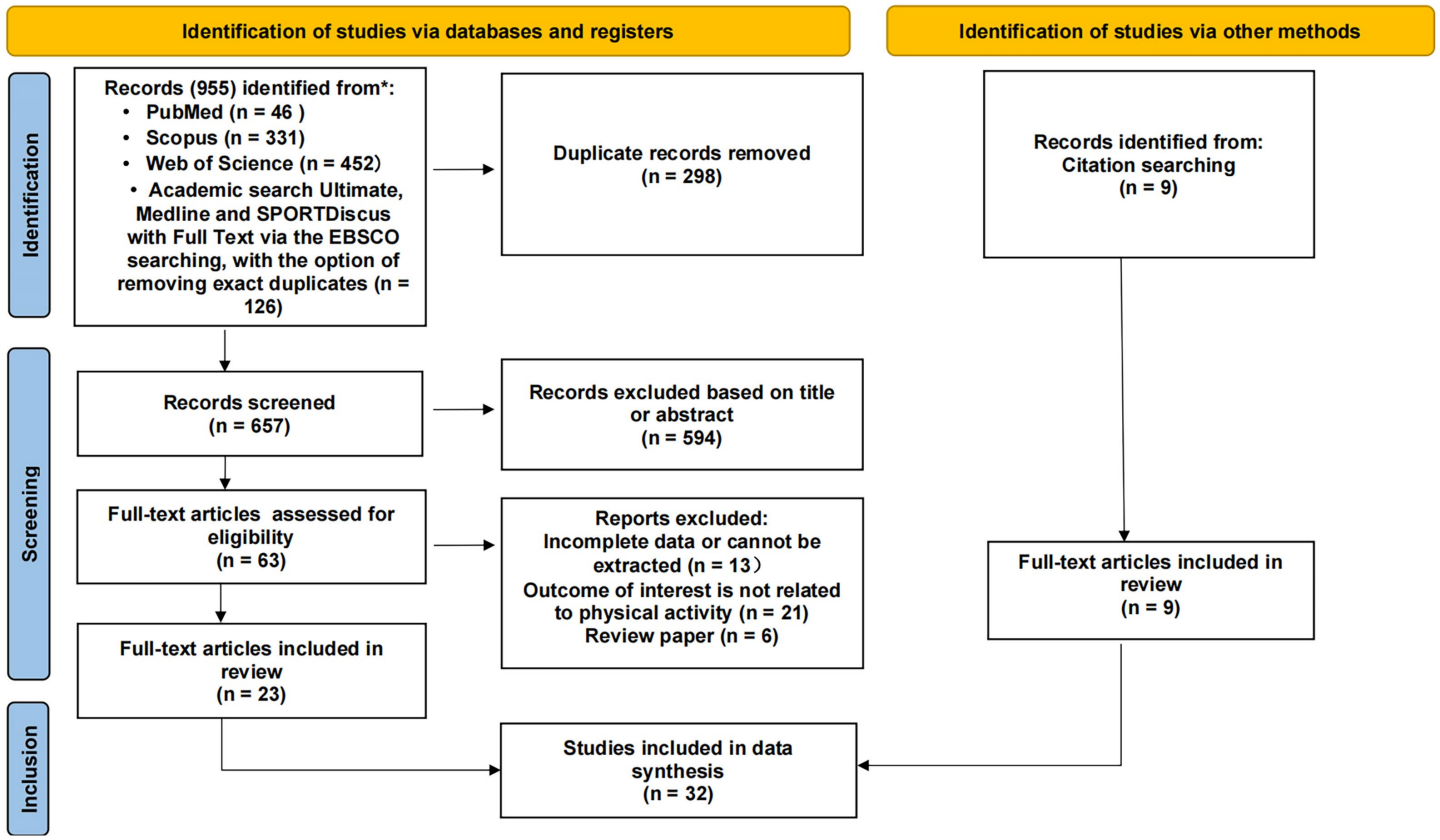
Flow diagram of study selection.

The date of the last search was 15 September 2022. Figure 1 shows the PRISMA diagram of the article screening process. The following inclusion and exclusion criteria were used to identify the eligible articles for review, and only empirical research articles were considered: Inclusion criteria were: (1) full text was available; (2) pregnant women were research participants; (3) a measurement or interview of PA (including MVPA) as the dependent outcome and examined the statistical associations with certain factors was reported; and (4) published in English-language, in scholarly (peer-reviewed) journals.

Exclusion criteria included research: (1) taking women with any disabilities or illnesses that could lower their ability in terms of bodily movement as the study population; (2) focusing on nutritional interventions or healthy eating; (3) involving a survey of parturient women; and (4) published only as an abstract, a comment, or a review due to a lack of data for extraction (but reference lists were checked for relevant studies).

Data extraction: Two independent researchers (SJ, MP) separately searched the databases and assessed the titles and abstracts of articles to determine the initial inclusions. The full texts were then assessed against the inclusion and exclusion criteria to finalize the articles eligible for inclusion in the review. Later, the two researchers independently analyzed all the articles using the extraction tables. If discrepancies were found and could not be resolved between the two researchers, a third researcher (AS) was invited to finalize the assessment.

## 4 Results

After removing duplicates and papers irrelevant to the selected topic (judging by the abstracts), 23 papers were finally included in the analysis (24–46). Based on the reference lists presented in these papers, additional 9 studies were included (47–55), and a total of 32 articles were at last included in the analysis (24–55) (Figure 1). Data including the author, date, number of people surveyed, research type, data collection method, country, and levels of SEM were extracted. To better understand the differences between factors influencing pregnant women’s overall PA and MVPA, articles covering the issue of MVPA were indicated for additional analysis. Based on the model established by McLeroy et al. (22), PA behavior is determined or affected by above mentioned five levels or groups of factors. If the study involved relevant factors, it was marked as √. The information is summarized in Table 1.

**Table 1:** Summary of studies on the factors influencing physical activity in pregnant women.

First author and year of publication	The number of women involved	Research type	Collection method	Country	Levels of SEM						
					Level 1			Level 2	Level 3	Level 4	Level 5
					Factors 1^1^	Factors 2	Factors 3				
Evenson K. R., 2004 (47)	1979	Quantitative	Tele-phone interview	United States	√/			√			
Schmidt M. D., 2006 (24)	233	Qualitative and quantitative	Questionnaire and interview	United States	√/√			√			
Chasan-Taber L., 2007 (48)	782	Quantitative	Interview	United States	√/√	√		√			
Evenson K. R., 2009 (25)	1,535	Qualitative and quantitative	Telephone interview	United States	√/√	√	√	√	√	√	√
Lynch K. E., 2012 (26)	903	Quantitative	Questionnaire	United States	/√	√		√			
Muzigaba M., 2014 (27)	34	Qualitative	Interview	South Africa	√/√	√	√	√	√	√	
Padmapriya N., 2015 (28)	1,171	Qualitative and quantitative	Interview	Singapore	√/√	√	√	√			
MRH Van Mulken, 2016 (29)	30	Qualitative	Telephone interview	Australia	/√	√	√	√	√	√	
Richardsen K. R., 2016 (30) (MVPA)	555	Qualitative and quantitative	Face-to-face interview	Norway	√/√	√		√			
Richardsen K. R., 2016 (31) (MVPA)	709	Qualitative and quantitative	Face-to-face interview and recorded PA data	Norway	√/		√			√	
Merkx A., 2017 (32)	455	Quantitative	Questionnaire	Netherlands	√/√	√	√		√	√	
Flannery C., 2018 (33)	22	Qualitative	Interview	Ireland	√/√	√	√	√	√	√	√
Rabiepoor S., 2019 (49)	325	Qualitative and quantitative	Questionnaire and interview	Iran	√/√	√	√	√			
Xiang M., 2019 (50) (MVPA)	1,077	Quantitative	Questionnaire	China	√/√	√					
Fathnezhad-Kazemi A., 2019 (51)	32	Qualitative	Interview	Iran	√/√	√	√	√	√	√	√
Hailemariam T. T., 2020 (34)	299	Quantitative	Questionnaire	Ethiopia	√/		√	√	√		
Walasik I., 2020 (35)	9,345	Quantitative	Questionnaire	Poland	√/√	√	√	√	√	√	
Okafor U. B., 2020 (36)	1,082	Qualitative and quantitative	Interview	South Africa	√/√	√			√		
Zhu G., 2020 (52)	746	Quantitative	Questionnaire	China	/√		√	√		√	
Silva V. R., 2021 (37)	3,580	Qualitative and quantitative	Face-to-face interview	Brazil	√/				√	√	
Kershaw K. N., 2021 (38) (MVPA)	8,362	Quantitative	Interview and measurement	United States	√/				√	√	
Ahmadi K., 2021 (39)	300	Quantitative	Questionnaire	Iran	√/√	√		√	√	√	
Jones M. A., 2021 (40) (MVPA)	127	Quantitative	Questionnaire and interview	United States	√/						
Lü Y., 2021 (41)	2,485	Quantitative	Questionnaire	China	√/√	√					
Grenier L. N., 2021 (53)	66	Qualitative	Face-to-face interview	United States	√/√	√	√	√	√	√	
Baena-García L., 2021 (54)	134	Quantitative	Objective measure	Spain	√/√			√			
Addis A., 2022 (42)	333	Qualitative	Face-to-face interview	Ethiopia	√/√		√	√	√		
Syed Nor S. F., 2022 (43)	316	Quantitative	Questionnaire	Malaysia	√/√		√	√		√	
Shum K. W., 2022 (44)	22	Qualitative	Semi-structured interview	Singapore	/√	√	√	√	√	√	
Kianfard L., 2022 (45) (MVPA)	40	Qualitative	Interview	Iran	√/	√	√	√	√	√	
Beyene M. M., 2022 (46)	410	Quantitative	Questionnaire	Ethiopia	√/	√	√	√	√	√	
Sparks J. R., 2022 (55) (MVPA)	431	Qualitative and quantitative	REDCap and interview	United States	√/√	√	√	√	√	√	
Total	37,920	9	21	14	28/24	21	19	24	18	18	3
		16	13		32						
		7	2								

SEM, socio-ecological model; MVPA, moderate-to-vigorous intensity physical activity; REDCap, Research Electronic Data Capture; Level 1, Personal level; Factors 1, socio-demographic and biological factors; Factors 2, behavioral factors; Factors 3, Psychological factors; Level 2, Interpersonal level; Level 3, Organization level; Level 4, Community level; Level 5, Public policy level. ^1^In the factors 1, on the left side refers to socio-demographic factors, and on the right, the “/” refers to biological factors.

### 4.1 Study characteristics

A summary of the characteristics of the 32 papers is given in Table 1. The publication period ranged from 2004 to 2022, with 20 (62.5%) published after 2019. We found results related to MVPA in pregnant women (30, 31, 38, 40, 45, 50, 55) in 7 papers. The sample sizes ranged from 22 to 9,345 participants, for a total of 37,920. The types of studies included mixed (9), quantitative (16), and qualitative (7) methods. The data were collected through interviews as well as questionnaires, and prospectively followed. Interview (21) and questionnaire (13) were the most popular methods, with only one other collection method. A total of 14 countries, including developed and developing countries, were involved. Relative studies contained different contents on a personal level. In addition, 28 papers included socio-demographic factors (24, 25, 27, 28, 30–43, 45–51, 53–55); 24 included biological factors (24–30, 32, 33, 35, 36, 39, 41–44, 48–55); 21 included behavioral factors (25–30, 32, 33, 35, 36, 39, 41, 44–46, 48–51, 53, 55); and 19 included psychological factors (25, 27–29, 31–35, 42–46, 49, 51–53, 55), 32 in total, all of which involved the personal level. There were 24 articles analyzing the interpersonal level (*N* = 24) (24–30, 33–35, 39, 42–49, 51–55); 18 analyzing the organization level (*N* = 18) (25, 27, 29, 32–39, 42, 44–46, 51, 53, 55); 17 articles included factors related to the community level (*N* = 17) (25, 27, 29, 31–33, 35, 37–39, 44–46, 51–53, 55); and only 3 articles included factors related the policy level (*N* = 3) (25, 33, 51).

### 4.2 Factors of physical activity during pregnancy in SEM

Tables 2–5 summarize papers containing the factors of PA during pregnancy referring to the five levels of SEM. In these papers we could find whether the identified association is a facilitator or a barrier. The direction of the association is expressed by a facilitator “+” or a barrier “−”. Relevant studies have different reference standards for the same factor (for example, the factor of knowledge and skills is a facilitator factor, while that of a lack of knowledge and skills is a barrier factor). To better analyze the factors of PA during pregnancy we also used following labeling: no association (coded with“0”) and as an inconclusive finding (coded with “?”).

**Table 2:** Summary of studies (*n* = 32) on personal level of socio-ecological model (SEM) influencing physical activity during pregnancy.

Level 1	Study reference number				Total number			
	Facilitator (+)	Barrier (−)	No association (0)	?	+	−	0	?
					69	94	37	5
Socio-demographic and biological factors					39	47	30	2
Age	(24)^1,*^, (34)^2,*^, (38)^1,*^, (47)^2^, (48)^1,*^	(36)^2^	(28, 32, 37), (40)^*^, (41)^2^, (43, 54, 55)		5	1	8	
Ethnicity	(24), (31)^*^, (40)^*^, (41)^3,*^,	(30, 38)^*^	(28, 37, 47)	(27, 33)	4	2	3	2
Education	(24), (30)^*^, (34, 37, 39), (40)^*^, (41–43, 47–49, 51)	(32)^a^, (35)^a^, (36)^a^, (43)^a^, (46)^a^, (54)^a^	(28, 50), (55)^*^		13	6	3	
Work	(34), (40, 45)^*^, (46, 49), (50)^*^	(25, 33), (36, 45)^*,b^, (53), (50)^*,b^, (51)	(28, 41, 43, 47, 54)		6	7	5	
Married status	(34), (40)^*^		(28, 54, 47), (55)^*^		2		4	
Income	(24, 39, 48)	(27)^*^, (33)^*^, (39), (45)^*^, (51), (53)^*^	(41)		3	6	1	
SES (family income)		(28)^a^, (51)^a^				2		
Parity	(24, 26, 42, 49, 54)	(29)^c^, (35), (36)^c^, (39), (43)^c^	(41)^d^		5	5	1	
Pregnancy discomforts		(25, 27, 28, 32, 33, 35, 43, 44, 51–53), (55)^*^				12		
BMI	(48)^*^	(25)^*^, (26)^*^, (30)^*^, (35)^a^, (43), (51)	(28, 41, 54), (50, 55)^*^		1	6	5	
Behavioral factors					12	20	5	0
Smoking		(41)	(48)			1	1	
Previous physical activity	(26, 29, 33, 35, 39, 41, 46, 48), (50)^*^, (53)	(28), (32), (36)^d,*^, (39)^d^, (50)^d,*^	(30)^*^, (44)		10	5	2	
Knowledge and skills	(29), (49)	(27)^d^, (33)^d^, (44)^d,*^, (45)^d^, (46)^d^, (53)^d^, (55)^d,*^			2	7		
Diet		(45)^*^	(50)^*^, (51)			1	2	
Lack of time		(25, 27, 33, 39, 44, 53)				6		
Psychological factors					18	27	2	3
Physical activity attitude	(33), (42), (45)^*^, (51), (52)	(46)^d^	(44)	(27), (32)	5	1	1	2
Physical activity intention	(42)	(46)^d^			1	1		
Perceived benefits of physical activity	(29, 33, 35, 43, 46, 49, 51), (55)^*^	(27)^d^, (29)^d^, (45)^*,d^, (52)^d^	(44)		8	4	1	
Barriers to physical activity		(35), (51), (55)^*^				3		
Perceived behavioral control	(42, 52)	(28)^d^			2	1		
Motivation/goal	(27, 33)	(25)^d^, (51)^d^, (53)^d^, (55)^*,d^		(32)	2	4		1
Safety concerns		(25, 27, 29), (31)^*^, (33, 34, 44), (45)^*^, (46, 49, 51, 53), (55)^*^				13		

Level 1, Personal level; BMI, body mass index; SES, socio-economic status; “+”, facilitator; “−”, barrier; “0”, no association; “?”, indeterminate association; ^a^The factor is a barrier when is low (such as: the education is a barrier when is low), ^b^The work is a barrier when is lose, ^c^The parity is first, ^d^The factor is a barrier when is lack (such as: motivation/goal is a barrier when is lack). ^1^older age, ^2^younger age, ^3^regions, *relates to MVPA.

**Table 3:** Summary of studies (*n* = 24) on interpersonal level of socio-ecological model (SEM) influencing physical activity during pregnancy.

Level 2	Study reference number				Total number			
	Facilitator (+)	Barrier (−)	No association (0)	?	+	−	0	?
					25	29	2	1
Family	(42, 44, 51, 53)	(29, 33, 35, 39, 44), (45)^*^, (51)	(27)	(53)	4	7	1	1
Colleague/Friend	(33, 44), (45)^*^	(29), (30)^*^			3	2		
Public	(44)	(29, 35), (45)^*^, (51, 53), (55)^*^			1	6		
Having children	(24, 26, 28, 34, 39, 43, 48, 49)	(25, 27), (30)^*^, (33, 44, 51, 52–54), (55)^*^	(47)		8	10	1	
Having a husband /partner	(27, 29, 33, 34, 44), (45)^*^, (46, 51, 53)	(39), (45)^*^, (46, 51)			9	4		

Level 2, Interpersonal level; “+”, facilitator; “−”, barrier; “0”, no association; “?”, indeterminate association; ^*^relates to MVPA.

**Table 4:** Summary of studies (*n* = 18) on organizational level of socio-ecological model (SEM) influencing physical activity during pregnancy.

Level 3	Study reference number				Total number			
	Facilitator (+)	Barrier (−)	No association 0	?	+	−	0	?
					16	13		
Health care providers	(27, 29, 34–37, 39, 42, 44), (45)^*^, (53)	(25), (27)^a^, (29)^a^, (32)^a^, (33)^a^, (36)^a^, (44)^a^, (45)^*^, (46), (51)^a^, (53)^a^, (55)^*,a^			11	12		
Nutritionists	(53)				1			
Dietitians	(53)				1			
Physical activity Consultant	(29, 53), (55)^*^	(38)^*,b^			3	1		

Level 3, Organization level; “+”, facilitator; “−”, barrier; “0”, no association; “?”, indeterminate association; ^a^The health care providers is a barrier when is lack; ^b^the physical activity consultant is a barrier when is lack; ^*^relates to MVPA.

**Table 5:** Summary of studies on community (*n* = 17) and public policy (*n* = 3) levels of socio-ecological model (SEM) influencing physical activity during pregnancy.

Level 4	Study reference number				Total number			
	Facilitator (+)	Barrier (−)	No association 0	?	+	−	0	?
					20	20	4	1
Information and resources	(32), (35), (44), (53)	(27)^a^, (32)^a^, (33)^a^, (45)^*,a^, (46)^a^, (51)^a^, (52)^a^, (53)^a^, (55)^*,a^			4	9		
Appropriate facilities	(27), (31)^*^, (37), (38)^*^, (51)	(27)^a^, (33)^a^, (45)^*,a^, (51)^a^		(53)	5	4		1
Living environment	(31)^*^, (38)^*^	(39), (51)	(37)		2	2	1	
Neighborhood safe	(31)^*^	(27)^a^, (45)^*,a^	(25)		1	2	1	
Media	(29), (32), (35), (45)^*^, (46), (51), (52), (53)	(27)^a^	(38)^*^		8	1	1	
Level 5		(33)^a^, (51)^a^	(25)			2	1	

Level 4, Community level; Level 5, Public policy level; “+”, facilitator; “−”, barrier; “0”, no association; “?”, indeterminate association; ^a^The factor is a barrier when is lack (such as: the public police is a barrier when is lack); ^*^relates to MVPA.

#### 4.2.1 Personal level (32 papers)

As shown in Table 2, personal factors include three aspects, involving 22 factors in total. In terms of socio-demographic and biological factors, a total of 10 factors associated with PA in pregnancy were presented. In 8 papers age of pregnant women tended to have no association with their PA level. There were also studies (*N* = 3 papers) in which older age had a promoting effect, while the effect of a younger age had diverse explanations. Being older appears to be associated with higher MVPA compliance (*N* = 3 papers). Some studies (*N* = 13 papers) considered high education a facilitator, and others (*N* = 6 papers) considered low education as a barrier. Higher education facilitated MVPA (*N* = 2 papers). Pregnancy discomforts were considered a barrier (*N* = 12 papers). In addition, authors considered high income as a facilitator (*N* = 3 papers) and regarded low income as a barrier (*N* = 6 papers). The first birth was considered a barrier (*N* = 3 papers), while parity was considered a facilitator (*N* = 5 papers) and a barrier (*N* = 2 papers). Regarding other factors such as ethnicity, work, marital status, and BMI, the results were not significantly different and remained controversial. There were differences in MVPA during pregnancy among different ethnic groups (*N* = 4 papers). Physical occupational work was related with higher amount of MVPA (*N* = 4 papers). The socioeconomic status (SES) was considered a barrier of PA during pregnancy (*N* = 2 paper).

There were five behavioral factors associated with PA during pregnancy, with the knowledge and skills on PA as a facilitator (*N* = 2 papers), a lack of knowledge and skills as a barrier (*N* = 7 papers), and a lack of time as a barrier (*N* = 6 papers). Some authors considered previous PA as a facilitator (*N* = 10 papers) and previous lack of PA as a barrier (*N* = 3 papers). Interestingly, other authors found that previous PA was a barrier (*N* = 2 papers), and in two papers there was an indeterminate association (*N* = 2 papers) between previous PA and PA during pregnancy. There were fewer studies about smoking (*N* = 2 papers) and diet (*N* = 3 papers).

There were 7 psychological factors associated with PA during pregnancy. PA attitude (*N* = 5 papers) and the perceived benefits of PA (*N* = 8 papers) were considered facilitators. The lack of perceived benefits of PA was considered a barrier (*N* = 4 papers). In three papers the authors mentioned specific barriers to PA (e.g., fear, anxiety, shame, exercise-induced fatigue, discomfort, and other perceptual disorders; *N* = 3 papers). Lack of motivation/goal was considered a barrier (*N* = 4 papers). Safety concerns were the main barrier to PA in pregnancy, mentioned in 13 studies.

#### 4.2.2 Interpersonal level (24 papers)

Table 3 summarizes the factors at the interpersonal level. Some studies considered family a facilitator (*N* = 4 papers) and its lack a barrier (*N* = 7 papers). Colleague/friend was considered a facilitator (*N* = 3 papers) and a barrier (*N* = 2 papers). Some authors identified the public as a barrier (*N* = 6 papers). Having children was considered a facilitator (*N* = 8 papers) and a barrier (*N* = 10 papers). Having a husband/partner was considered a facilitator (*N* = 9 papers) and a barrier (*N* = 4 papers).

#### 4.2.3 Organizational level (18 papers)

Table 4 summarizes the factors at the organizational level. There were four external social support organizational factors for PA during pregnancy, with health care providers as the main influencing factors. Health care providers were considered a facilitator (*N* = 11 papers) and, interestingly, also as a barrier (*N* = 3 papers). In addition, a lack of healthcare provider support was considered a barrier (*N* = 9 papers).

#### 4.2.4 Community (17 papers) and public policy (3 papers) level

Table 5 summarizes factors at the community and public policy levels. There were five community and policy factors involved in PA during pregnancy. Information and resources were considered a facilitator (*N* = 4 papers), while a lack of information was considered a barrier (*N* = 9 papers). Appropriate facilities was considered a facilitator (*N* = 5 papers), and a lack of appropriate facilities was a barrier (*N* = 4 papers). In one study the authors mentioned a continuous positive correlation between good access to recreation sites and MVPA throughout pregnancy. The access to mass media was considered a facilitator (*N* = 8 papers), and living environment was considered both a facilitator (*N* = 2 papers) and a barrier (*N* = 2 papers). There were only a few studies on neighborhood safety (*N* = 4 papers) and the public policy level (N = 3 papers).

## 5 Discussion

This systematic review aims to examine existing studies on factors affecting PA in pregnancy, referring to the SEM developed by McLeroy et al. (22). We wanted to explore barriers and facilitators of PA in pregnant women, using the five levels of SEM. In the interpretation of our data it must be taken into account, that the research types are mainly quantitative (*N* = 16 papers), and the sample for the qualitative evaluation is relatively small (*N* = 7 papers). It was found that different research types might lead to different results, while the combination of qualitative and quantitative types (*N* = 9 papers) might lead to a more accurate investigation of influencing factors. In the analyzed material, all studies involved the personal level, and very few concerned the policy level. Through literature analysis, it was also found that there were some differences in the factors affecting overall PA and MVPA during pregnancy, which need to be further verified. It must be underlined that one of the main barrier to PA during pregnancy were the safety concerns.

Personal factors included socio-demographic and biological, behavioral, and psychological factors. Age was not considered to be associated with PA in pregnant women (28, 32, 37, 40, 41, 43, 54, 55) by most researchers. Being older appears to be associated with higher MVPA compliance (24, 38, 48). Our observations show that the age of pregnant women should be taken into account when planning PA interventions. In terms of different ethnicities, the research views were controversial. There were differences in MVPA during pregnancy among different ethnic groups (30, 31, 38, 40). There might also be differences between different parts of the same country (41). Considering that all the studies analyzed the outcomes from one country, ethnic differences should still be considered when implementing the guidelines and policies supporting PA in pregnancy. Education was significantly associated with PA during pregnancy, and higher education promoted PA (24, 34, 37, 39, 41–43, 47–49, 51) and MVPA (30, 40). High income was also a PA facilitator (24, 39, 48). We can assume that higher education probably is associated with higher income and also with intellectual professional activity. There were some debates about the impact of work on PA during pregnancy. Some authors claim that heavy physical work can have negative impact on the progression of pregnancy. Nevertheless, it increases the amount of MVPA (36, 40, 45, 50). However, since exercise was a small part of maternal activity, and work affected PA duration (45), a balance should be struck in future interventions focused on the implementation of PA programs in women who perform physical occupational work.

Opinions vary widely on the impact of pre-pregnancy PA on PA during pregnancy, with one survey finding that previously active participants expressed that they did not continue their active lifestyle during pregnancy (44). It was even found that women with higher levels of PA before pregnancy were more likely to reduce PA (28, 32). The main interpretations for this result were that the pre-pregnancy PA was self-reported so there might be memory bias (30, 44), and that the pregnant women were recommended to stop their favorite exercise or other activity (32) by family and even care providers. In contradiction to these outcomes, previous PA was seen as a facilitator in other studies (26, 29, 33, 35, 39, 41, 46, 48, 50, 53), while inactivity before pregnancy was seen as a barrier (36, 39, 50). In one paper, there was a suggestion that PA intensity could be increased by encouraging more PA before pregnancy (40). Low education (32, 35, 36, 43, 46, 54), first parity (29, 36, 43), lack of knowledge and skills (27, 33, 44–46, 53, 55), pregnancy symptoms (25, 27, 28, 32, 33, 35, 43, 44, 51–53, 55), lack of time (25, 27, 33, 39, 44, 53), low income (27, 33, 39, 45, 51, 53), lack of motivation/goal (25, 51, 53, 55), and safety concerns (25, 27, 29, 31, 33, 34, 44–46, 49, 51, 53, 55) were all identified by the researchers as barriers. The lack of time mainly came from family commitments (44), while low education, first parity, and lack of knowledge and skills were all related to safety concerns. Fewer of the included articles addressed diet and smoking. It is very worrying that, although some pregnant women showed positive attitudes toward PA and agreed with its benefits, most did not engage in PA (44). There was a discord between positive attitudes toward PA and actual behaviors (44). In future PA interventions, more attention should be paid to populations with low education, first parity, lack of knowledge, and low income, and further research should be conducted on the way to effectively utilize the positive attitudes and perceptions of pregnant women. What is more, evidence-based educational programs on various forms and intensities of PA, including higher intensity exercise (6) should be implemented.

Analysis of the interpersonal level, which included family, colleagues/ friends, and husband revealed that these factors could both promote and be a barrier to PA in pregnant women. A commonly reported facilitators were “social influences,” which included encouragement of PA by family and friends. Women’s partners or husbands were the most influential factor, and women enjoyed meeting other pregnant women and expressed interest in PA classes tailored to pregnancy (33). Having an active spouse before pregnancy was identified as the strongest predictor of performing moderate-intensity to vigorous-intensity PA during pregnancy (39). Additionally, emotional support from family and friends was commonly mentioned as one of the motivators for undertaking PA during pregnancy (44). Studies also found that during pregnancy, women were discouraged from PA by people at work and the gym, as well as family and acquaintances (29). A study observed that participants lacking encouragement from mothers and mothers-in-law tended to not engage in PA (56). Similarly, pregnant women received most of their advice on PA from their families, friends, or media (57). It was also reported that conflicting advice regarding PA from healthcare professionals and family members was confusing (44). The public was unanimously cited as a barrier factor (29, 35, 45, 51, 53, 55), and physically active women were more often criticized than praised for being active during pregnancy. Women commonly felt stared at, avoided, and treated differently during pregnancy, and often felt treated as if they were infirm or disabled, and that their pregnancy was viewed as a disease (29). Having a child was somewhat controversial; it was seen as a barrier mainly due to the need to care for other children and the limited exercise time available, which made it challenging to perform physical activities outside a daily routine (25, 27, 30, 33, 44, 51–55). In studies about having children as a facilitator, results were mainly derived from data; however, mainly qualitative analysis provided results indicating it was a barrier. Further analysis of the impact of different research types on the results is still needed. In future PA in pregnant women interventions, the family and husband/partner should be very important facilitators, and it is necessary to get the family and husband involved.

Third, at the organizational level, our analysis focused on the role of health care providers, and the results were consistent. The health care providers were the facilitating factor for engagement in PA during pregnancy (27, 29, 34–37, 39, 42, 44, 45, 53). The lack of support from health care providers was a barrier to pregnant women to be physically active (27, 29, 32, 33, 36, 44, 51, 53, 55). Surprisingly, in three papers we found that the health care providers themselves were identified as a barrier. We may assume that their conservative approach to prenatal PA may discourage their patients from performing PA. Related studies found that educating pregnant women about PA was not a priority for healthcare professionals who provided prenatal care during routine antenatal visits. As a result, many pregnant women did not receive information and advice about PA from healthcare providers (58, 59). Informational support from healthcare providers could have a significant influence on the views and decisions of pregnant women (59). In addition, nutritionists (53), dieticians (53), and PA consultants (29, 53, 55) might promote PA in pregnant women. It could also be observed that pregnant women’s dependence on the advice of health care providers was related to the lack of participation of other organization members (which might be the specialists indicated above). In the future, more scientific research institutions, schools, fitness operators, and health promotion agencies should participate in PA intervention guidance for pregnant women. Importantly, a tailored educational programs for health and exercise professionals should be developed and implemented to prepare them to properly support pregnant women in the engagement in PA.

At the community level, both information and resources were considered a facilitator (32, 35, 44, 53), while the lack of information and resources was a barrier of PA in pregnant women (27, 32, 33, 45, 46, 51–53, 55). The analyzed studies revealed that participants were more likely to be active if they received sufficient information about PA during pregnancy (37, 44). Unfortunately, many participants also reported the lack of access to information (27, 33, 45, 53). The access to sport facilities was also a facilitator (27, 31, 37, 38, 51), while a lack of it was considered a barrier (27, 33, 45, 51). There was a continuous positive correlation between good objective access to recreation sites and MVPA throughout pregnancy (31). Research supported this view that high-quality PA was associated with the quality of PA amenities (60). Living environment was sometimes considered a facilitator (31, 38) and sometimes a barrier (39, 51). In a word, the physical attributes of neighborhoods are positively associated with PA (31). The access to mass media promoted PA in pregnant women (29, 32, 35, 45, 46, 51–53), and access to mass media and education were very important factors in raising public awareness (29). In future health education, it is very necessary to establish corresponding obstetrics lectures, new mass media platforms, and valid internet sites for education to provide information sources and enhance citizen consciousness.

Finally, in relation to the public policy level, there were only a few studies on PA policies for pregnant women, and policies were not a major concern for pregnant women (25). However, as the external driving force that influenced an individual’s participation in physical activities, socio-ecological theory emphasized that regulations, educational policies, public health policies, etc. at the outermost levels of policy had a pronounced impact on individual behavior (22). At the same time, most prenatal PA interventions were based on recommendations from national and international organizations (55). Yet, as reported by the WHO on October 19, 2022, data from 194 countries showed that overall, less than 50% of countries had a national PA policy, of which less than 40% were being implemented (61). It was also evident from this review that policies did not seem to be working as they should, and this supported the view of the WHO that there were gaps in the formulation of policies and serious gaps in their implementation concerning PA. Policies played a guiding role for organizations and individuals and guaranteed cooperation between different regions, sectors, and groups. In the future, countries should refer to WHO policy recommendations in the global status report on PA 2022, increase their levels of participation across four strategic policy areas, including active societies, active environments, active people, and active systems (62) and enhance the policy drive for individual behavior.

Pregnant women face more obstacles to their PA compared to non-pregnant populations. As a result, they cannot comply with PA recommendations, unless these obstacles are overcome (55). The lack of organizational and policy support is an important factor that makes it difficult for pregnant women to engage in PA despite their willingness. A study has shown that implementing a PA plan that meets the recommended level of PA may be more effective if prescribed individually by an appropriate specialist or trained clinician (55). PA is the key to improving health and addressing non-communicable diseases (62). Therefore, overcoming barriers to PA requires a deep integration of PA and medicine. With advancements in technology, mobile applications and digital technologies provide opportunities for real-time interaction, information sharing, and multisectoral collaboration (63). In this case, governments should join forces with relevant organizations to increase advocacy and knowledge and consolidate resources to create a more supportive environment through a government-led, multisectoral collaborative approach to health interventions. At the national level, efforts should be made to establish a digital technology-based, government-led, multi-sector cooperative health integration intervention system, thereby creating a more supportive and friendly environment for the PA of pregnant women.

Our literature review is of high practical value. In particular, due to the fact that there is a very low level of PA during pregnancy in different populations worldwide (64). For example, a study done in Ethiopia (2019), a low-income country, reported a physical inactivity prevalence of 21.9% (65). One study conducted in the United States (2013) identified that 31% of pregnant women reported engaging in mild-intensity activities, 38% in moderate-intensity, and 32% in vigorous-intensity PA (66). This figure was even lower in a study from Brazil (2010), where only 4.7% of pregnant women were physically active (67). Only one-fifth of pregnant women in Ireland (2011) met the recommended guidelines, and over 10% reported no PA (68). A cross-sectional study (2014) among urban Chinese women reported that 74.4% of total participants reduced PA during pregnancy (69). A Shanghai study (2022) found that only 2.8% of pregnant women achieved the level of prenatal PA recommended by the international guidelines (70). An adequate level of PA in Iran (2010) was found to be 39% (71), in Norway (2020) it was 14.6% (72), in India (2015) 18% (73), and in Nigeria (2014) 10.2% (74). These numbers prove the need to take systemic measures to promote PA during pregnancy in various countries, including underdeveloped countries.

This review study has its limitations. First, one criterion for inclusion in the analysis is the publication of papers in English. There may be other studies on maternal PA influencing factors available in other languages. Second, since much of the literature is qualitative, relevant factors are not directly reflected in the results and conclusions, which made potential bias in our review analysis. Third, there are other types of socio-ecological models or theories (75). Although this review followed a rigorous, systematic protocol, given the ontological and epistemological assumptions inherent to configurative reviews (76), other studies and reviews that followed different SEM or theories might have addressed the factors differently. In consequence, they may not result in the same conclusions.

## 6 Conclusion

Through this systematic review, it was found that SEM can provide a wide-ranging overview of factors that influence PA in pregnant women. Nevertheless, a more comprehensive system of factors revision is needed, where a more accurate approach combining qualitative and quantitative methods will be used. The factors that positively influenced PA in pregnant women were: higher levels of education, knowledge and skills, as well as access to mass media. Conversely, lower levels of education, lack of knowledge and skills, low income, pregnancy discomforts, limited time, safety concerns, and societal perceptions acted as barriers. Additionally, family, colleagues/friends, and partners could either support or hinder PA. Factors affecting maternal overall PA are somewhat different from those affecting MVPA.

Safety concerns are the main barriers to PA in pregnant women. Therefore, the solutions addressing safety concerns should be a significant issue in promoting maternal PA. What is more, pregnant women receive little organizational and policy support and are exposed to a lack of external drivers to be physically active. There is an urgent need to provide accessible information and resource systems for pregnant women. Since most pregnant women are motivated to engage in PA and susceptible to family advice, interventions should not be limited only to pregnant women, but should involve a family member, especially partners. For pregnant women themselves, physical activity or exercise prescriptions tailored individually by appropriate specialists or trained clinicians may be the most effective means to help them meet PA guidelines, all of which need to be supported by government policies.

## Author contributions

JS and AS contributed to the conception and design of the study. JS, AS, MP, and AW performed data collection and analysis. JS wrote the first draft of the manuscript. AS and MP screened, reviewed, and revised the manuscript. All authors contributed to the article and approved the submitted version.
