# Supplementary material for: A socio-ecological model of factors influencing physical activity in pregnant women: a systematic review
Source: Front Public Health. 2023 Nov 20;11:1232625. doi: 10.3389/fpubh.2023.1232625 (PMC10694207; doi:10.3389/fpubh.2023.1232625)
Supplement: Supplementary file 1 [file Data_Sheet_1.docx]

Supplementary Material

# Search strategy

**PubMed:** 46

#4 #1 AND #2 AND #3 46

#3 "pregnant women"[All Fields] 115,868

#2 "correlates"[All Fields] OR "determinants"[All Fields] OR "mediators"[All Fields] OR "associated factors"[All Fields] OR "psychosocial"[All Fields] OR "environment"[All Fields] 1,547,300

#1 "physical activity"[All Fields] OR ("exercise"[MeSH Terms] OR "exercise"[All Fields] OR "exercises"[All Fields] OR "exercise therapy"[MeSH Terms] OR ("exercise"[All Fields] AND "therapy"[All Fields]) OR "exercise therapy"[All Fields] OR "exercise s"[All Fields] OR "exercised"[All Fields] OR "exerciser"[All Fields] OR "exercisers"[All Fields] OR "exercising"[All Fields] OR ("fitness"[All Fields] OR "fitnesses"[All Fields])) OR "physical exercise"[All Fields] OR "sport"[All Fields] 736,894

Scopus: 331

#4 #1 AND #2 AND #3 331

#3 ALL(women) 1,891,803

#2 ALL(((((correlates) OR (determinants)) OR (mediators)) OR (associated factors)) OR (psychosocial)) OR (environment) 4,995,131

#1 ALL(((physical activity) OR (exercise or fitness)) OR (physical exercise)) OR (sport) 556,625

Web of Science: 452

#4 #1 AND #2 AND #3 452

#3 ALL(women) 2,289,646

#2 ALL(((((correlates) OR (determinants)) OR (mediators)) OR (associated factors)) OR (psychosocial)) OR (environment) 6,121,747

#1 ALL(((physical activity) OR (exercise or fitness)) OR (physical exercise)) OR (sport) 504,337

Ebsco: 126

TX (((physical activity) OR (exercise or fitness)) OR (physical exercise)) OR (sport) ) AND TX (((((correlates) OR (determinants)) OR (mediators)) OR (associated factors)) OR (psychosocial)) OR (environment)) AND TX (women) 126
