# Supplementary material for: A socio-ecological model of factors influencing physical activity in pregnant women: a systematic review
Source: Front Public Health. 2023 Nov 20;11:1232625. doi: 10.3389/fpubh.2023.1232625 (PMC10694207; doi:10.3389/fpubh.2023.1232625)
Supplement: Supplementary file 2 [file Data_Sheet_2.docx]

Supplementary Material

# Quality Appraisal

**Table 1.** MMAT qualitative studies quality appraisal results.

| Methodological quality criteria | Study Reference Number | | | | | | |
| --- | --- | --- | --- | --- | --- | --- | --- |
|  | 1 | 2 | 3 | 4 | 5 | 6 | 7 |
| 1.1. Is the qualitative approach appropriate to answer the research question? | Y | Y | Y | Y | Y | Y | Y |
| 1.2.Are the qualitative data collection methods adequate to address the research question? | Y | Y | Y | Y | Y | Y | Y |
| 1.3. Are the findings adequately derived from the data? | Y | Y | Y | Y | Y | Y | Y |
| 1.4. Is the interpretation of results sufficiently substantiated by data? | Y | Y | Y | Y | Y | Y | Y |
| 1.5.Is there coherence between qualitative data sources, collection, analysis and interpretation? | Y | Y | Y | Y | Y | Y | Y |
| Y = YES, N = NO, C = Cannot tell; Mixed Methods Appraisal Tool (MMAT); 1=Shum KW(2022)[44],2= MRH Van Mulken (2016)[29], 3=Muzigaba M(2014) [27], 4=Flannery C( 2018)[33], 5=Kianfard L (2022)[45](MVPA), 6=Grenier L N (2021)[53],7=Fathnezhad-Kazemi A(2019)[51] | | | | | | | |

**Table 2.** MMAT quantitative studies quality appraisal results.

| Category of study designs | Methodological quality criteria | Study Reference Number | | | | | | | | | | | | | |
| --- | --- | --- | --- | --- | --- | --- | --- | --- | --- | --- | --- | --- | --- | --- | --- |
|  |  | 8 | 9 | 10 | 11 | 12 | 13 | 14 | 15 | 16 | 17 | 18 | 19 | 20 | 21 |
| Quantitative  descriptive | 4.1. Is the sampling strategy relevant to address the research question? | Y | Y | Y | Y | Y | Y | Y | Y | Y | Y | Y | Y | Y | Y |
|  | 4.2. Is the sample representative of the target population? | Y | Y | Y | Y | Y | Y | Y | Y | Y | Y | Y | N | Y | Y |
|  | 4.3. Are the measurements appropriate? | Y | Y | Y | Y | Y | Y | Y | Y | Y | Y | Y | Y | Y | Y |
|  | 4.4. Is the risk of nonresponse bias low? | Y | Y | Y | Y | Y | Y | Y | Y | N | Y | Y | N | N | Y |
|  | 4.5. Is the statistical analysis appropriate to answer the research question? | Y | Y | Y | Y | Y | Y | Y | Y | Y | Y | Y | Y | Y | Y |
| Y = YES, N = NO, C = Cannot tell; Mixed Methods Appraisal Tool (MMAT);8=Hailemariam T T (2020)[34],9=Addis A (2022)[42],10=Walasik I (2020)[35], 11=Syed Nor S F (2022)[43],12=Merkx A(2017)[32],13=Lynch K E(2012)[26],14=Kershaw K N 2021[38] (MVPA),15=Ahmadi K(2021)[39],16=Jones M A (2021)[40]MVPA, 17=Lü Y (2021)[41],18=Beyene M M (2022)[46],19=Baena- García L (2021)[54],20=Xiang M (2019)[50](MVPA), 21=Zhu G (2020)[52] | | | | | | | | | | | | | | | |

**Table 3.** MMAT quantitative studies mixed methods appraisal results.

| Methodological quality criteria | Study Reference Number | | | | | | | | | | |
| --- | --- | --- | --- | --- | --- | --- | --- | --- | --- | --- | --- |
|  | 22 | 23 | 24 | 25 | 26 | 27 | 28 | 29 | 30 | 31 | 32 |
| 5.1. Is there an adequate rationale for using a mixed methods design to address the research question? | Y | Y | Y | Y | Y | Y | Y | Y | Y | Y | Y |
| 5.2. Are the different components of the study effectively integrated to answer the research question? | Y | Y | Y | Y | Y | Y | Y | Y | Y | Y | Y |
| 5.3. Are the results adequately brought together into overall interpretations? | Y | Y | Y | Y | Y | Y | Y | Y | Y | Y | Y |
| 5.4. Are divergences and inconsistencies between quantitative and qualitative results adequately addressed? | Y | Y | Y | Y | Y | Y | Y | Y | Y | Y | Y |
| 5.5. Do the different components of the study adhere to the quality criteria of each tradition of the methods involved? | N | Y | N | Y | Y | N | N | Y | N | Y | N |
| 22=Silva V R (2021)[37],23=Padmapriya N (2015)[28],24=Richardsen K R (2016)[30](MVPA),25= Okafor U B (2020)[36],26=Richardsen K R(2016)[31](MVPA), 27=Evenson K R (2009)[47], 28=Schmidt M D (2006)[24],29=Rabiepoor S (2019)[49], 30=Chasan-Taber L(2007)[48],31=Evenson K R(2004)[25],32=Sparks J R 2022[55](MVPA) | | | | | | | | | | | |
